# Role of PDZ-binding motif from West Nile virus NS5 protein on viral replication

**DOI:** 10.1038/s41598-021-82751-x

**Published:** 2021-02-05

**Authors:** Emilie Giraud, Chloé Otero del Val, Célia Caillet-Saguy, Nada Zehrouni, Cécile Khou, Joël Caillet, Yves Jacob, Nathalie Pardigon, Nicolas Wolff

**Affiliations:** 1 Unité Interactions Virus-Insectes, Institut Pasteur, Paris, France; 2 Unité Récepteurs-Canaux, Institut Pasteur, Paris, France; 3 Unité de Génétique Moléculaire des Virus ARN, Institut Pasteur, Paris, France; 4 UMR 8261, CNRS, Université de Paris, Institut de Biologie Physico-Chimique, 75005 Paris, France; 5 Unité de Recherche et d’Expertise Environnement et Risques Infectieux, Institut Pasteur, Paris, France

**Keywords:** Protein-protein interaction networks, West nile virus

## Abstract

West Nile virus (WNV) is a Flavivirus, which can cause febrile illness in humans that may progress to encephalitis. Like any other obligate intracellular pathogens, Flaviviruses hijack cellular protein functions as a strategy for sustaining their life cycle. Many cellular proteins display globular domain known as PDZ domain that interacts with PDZ-Binding Motifs (PBM) identified in many viral proteins. Thus, cellular PDZ-containing proteins are common targets during viral infection. The non-structural protein 5 (NS5) from WNV provides both RNA cap methyltransferase and RNA polymerase activities and is involved in viral replication but its interactions with host proteins remain poorly known. In this study, we demonstrate that the C-terminal PBM of WNV NS5 recognizes several human PDZ-containing proteins using both *in vitro* and *in cellulo* high-throughput methods. Furthermore, we constructed and assayed in cell culture WNV replicons where the PBM within NS5 was mutated. Our results demonstrate that the PBM of WNV NS5 is important in WNV replication. Moreover, we show that knockdown of the PDZ-containing proteins TJP1, PARD3, ARHGAP21 or SHANK2 results in the decrease of WNV replication in cells. Altogether, our data reveal that interactions between the PBM of NS5 and PDZ-containing proteins affect West Nile virus replication.

## Introduction

Arboviruses include numerous human and animal pathogens that are important global health threats responsible for arboviroses. Some arboviroses are the most serious worldwide infectious risks to the human nervous system^1^. Among them, Flaviviruses from the *Flaviviridae* family are transmitted by the bite of arthropods (mosquitoes or ticks), and are able to replicate both in invertebrate and vertebrate organisms. Tick born encephalitis virus (TBEV), West Nile virus (WNV) and Japanese encephalitis virus (JEV) are neurotropic Flaviviruses causing febrile illness in humans that may progress to encephalitis and even death in some cases.

Hijacking of cellular protein functions is a widely used strategy for viruses as they are obligate intracellular pathogens. Each step of the viral life cycle from entry to transmission is orchestrated through interactions with cellular proteins. All families of viruses manipulate the cell proteome by targeting key proteins involved in the control of cell homeostasis. Interactions mediated by short linear motifs (SLiMs) are ubiquitous in eukaryotic proteome^2^ and the adaptation of viruses to their environment could involve the extensive use of SLiM mimicry to subvert host functionality. PDZ-Binding Motifs (PBMs) are short SLiMs that interact with a large family of protein–protein interaction domains found in prokaryotes and eukaryotes called PDZ (PSD-95/Dlg/ZO-1), and are usually located at the extreme carboxyl terminus of proteins, although a few internal binding motifs have also been identified^3^. PBMs play a central role in cell signaling by mediating protein–protein interactions in complex networks such as establishment and maintenance of cell polarity. The PDZ-PBM interactome is therefore one of the most prominent instances of a SLiM-mediated protein interaction network serving key cell signaling purposes. PBMs were identified in proteins of many viruses, responsible for acute to chronic infection, illustrating that cellular PDZ proteins are common targets during viral infection^3^. Cellular PDZ/viral PBM interactions were shown as directly involved in viral pathogenicity of severe acute respiratory syndrome coronavirus (SARS-CoV) and of rabies virus as well as in the oncogenicity of human papillomavirus (HPV16)^4–6^. During infection, the viral proteins compete with the endogenous protein ligands through the binding to the PDZ domain of the host protein targets but can also affect the catalytic activity of signaling proteins^7^. Functional perturbations of cellular processes due to interactions between viral PBMs and cellular PDZ-containing proteins may improve the virus life cycle in the host and the dissemination to new hosts. Thus, targeting the PBM-PDZ interface could lead to novel antiviral therapies.

Internal and C-terminal PBMs are present in the non-structural (NS) proteins NS5 of Flaviviruses which provides the RNA cap methyltransferase (MTase) and the RNA-dependent RNA polymerase (RdRp) activities as well as suppression of type 1 interferon signaling^8^. Their association with PDZ-containing proteins has been demonstrated for TBEV and dengue virus (DV)^9,10^. TBEV NS5 contains a PBM in both the MTase and RdRp regions. The binding of the internal PBM located in the MTase to a PDZ domain located in the Scribble cellular protein inhibits IFN-mediated JAK-STAT signaling, countering innate immunity to the virus^11^. Results with DV NS5 also suggest an internal binding mechanism to target PDZ-containing protein ZO-1 (also known as TJP1)^9,11^ that may contribute to changes at the tight junctions affecting the trans-endothelial permeability^12^. WNV NS5 displays a typical class I PDZ binding motif (S/T-X-L/V/I) at the C-terminal PBM of its RdRp region (-TVL) as does TBEV (-SII)^10^, while any internal PBM corresponding to that of TBEV MTase is rather elusive. To carry out viral genome replication, Flavivirus assembles a replication complex, NS5 protein is one major component of this complex. It is highly probable that the PBM of WNV NS5 protein plays a role during infection, in particular during replication by interacting with host partners.

Here, we decipher the role of PBM from WNV NS5 in the viral life cycle. We show both *in vitro* and *in cellulo* that this PBM is fully functional and binds to several PDZ-containing cellular proteins using high-throughput interactomic studies. Mutations in the PBM of NS5 result in significantly reduced WNV replication *in vitro* after cell transfection and depletion of some of these PDZ-containing proteins also affects WNV replication.

## Results

### The PBM within NS5 impacts West Nile virus replication

**Figure 1:**
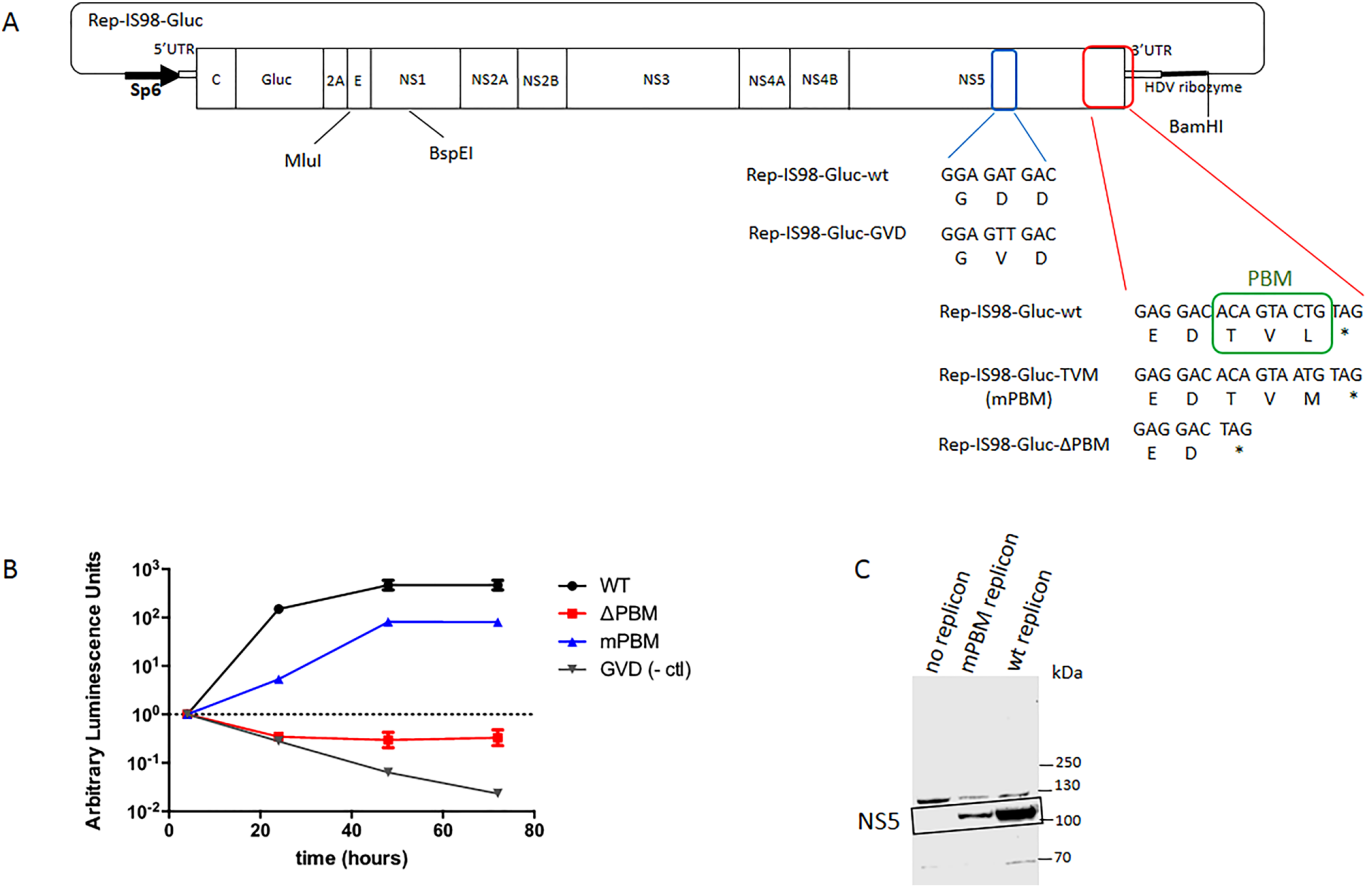
WNV replicon experiments. (**A**) Schematic construction of DNA based subgenomic WNV IS98 replicon models with mutations in the PBM of NS5. Rep-IS98-Gluc is a replicon containing the non-structural genes of WNV while the deleted structural genes were replaced by the non-secreted form of Gaussia luciferase (Gluc) reporter gene under the control of Sp6 promoter. The PBM sequence is indicated in green, a (−TVL) motif at the C-terminal extremity of NS5 (Rep-IS98-Gluc-wt). Three other replicons were constructed: a negative control replicon (Rep-IS98-Gluc-GVD), a replicon with a deletion of the three residues –TVL– (Rep-IS98-Gluc-∆PBM) and a mutagenized PBM in the replicon (Rep-IS98-Gluc-TVM: mPBM). (**B**). Replication of IS-98-Gluc replicons into BHK-21 cells. Representative replication curves after transfection into BHK-21 cells are plotted for replicons expressing different NS5 sequences: Rep-IS98-Gluc-wt (WT), Rep-IS98-Gluc-TVM (mBPM), Rep-IS98-Gluc-∆PBM (∆PBM) and a negative control replicon Rep-IS98-Gluc-GVD (GVD). The cells were lyzed for subsequent analysis post-transfection. Relative expression of luminescence was normalised to the luminescence obtained 4 h post-transfection. Average arbitrary luminescence units ± SEM is shown from 4 wells per group; experiments were done in triplicate. (**C**). Western blot showing NS5 protein levels in BHK-21 cells transfected with no replicon (medium only), wt replicon or mPMB replicon. Western blot of lysates from transfected BHK-21 cells at 48 h post electroporation of medium only (negative control/no replicon), mPBM replicon or wt replicon as indicated. The Western-blot is performed with the anti-NS5 antibody. Uncropped WB is shown in Supplementary Fig. 3.

The NS5 protein of some Flaviviruses are known to contain an internal and/or C-terminus PBM. The WNV NS5 protein contains a −TVL_COOH_ motif at the C-terminal extremity, corresponding to a PBM of class I with the motif S/T-X-Φ_COOH_ where X is any amino-acid, and Φ is a hydrophobic amino-acid (Fig. 1A). As NS5 of Flaviviruses provides RNA cap MTase and RdRp activities, we focused our efforts to determine whether the PBM of WNV NS5 affects virus replication. To evaluate the contribution of the PBM to WNV replication, we constructed a DNA based sub-genomic WNV replicon expressing the 5′ and 3′ UTR, the genomic region encoding all the non-structural proteins of WNV as well as the reporter gene GLuc, thus generating a luciferase expressing replicon (Rep-IS98-Gluc-wt) (Fig. 1A). We also constructed a negative control replicon coding for an inactive RdRp with a single mutation in the replication “pocket” on WNV NS5 (Rep-IS98-Gluc-GVD) and a replicon with a deletion of the three residues -TVL identified as the PBM motif (Rep-IS98-Gluc-∆PBM) (Fig. 1A). The replicons were transfected into BHK-21 cells and their replication rates were monitored using luciferase luminescence at different time points over 72 h post-transfection (Fig. 1B). The deletion of the nucleotidic sequence coding for the PBM (∆PBM) resulted in a drastic effect with an absence of replication of the WNV replicon (Fig. 1B). In order to confirm the role of the PBM sequence in the replication of WNV, a mutagenized PBM in the replicon was also designed (Rep-IS98-Gluc-TVM: mPBM). We introduced suitable mutations into the replicon taking care not to alter the RNA structure. Indeed, the genome of WNV is an RNA molecule displaying a single ORF flanked by untranslated regions (UTRs). These UTRs are structural conserved parts in the viral genome and play important roles in viral translation and replication. Optimal viral RNA structure at the 3′ UTR is required for efficient polymerase activity during WNV replication leading to viral production in cultured cells^13^. Introducing a mutation in the RNA sequence encoding a PBM located at the hinge between translated and untranslated regions has proved challenging since *cis*-acting RNA structures in the 3′-genome must be considered in WNV replication in cells in addition to RdRp activity. We did identify amino-acids and nucleotides required to maintain replication competence and replication efficiency as some PBM mutations in replicons abrogated replication, due to modifications of secondary structures of the 3′UTR. A functional replicon that conserved a proper predicted RNA structure while altering the PBM was thereby obtained using the mutation Leu to Met at the C-terminus of NS5. Thus, we introduced a mutation in the PBM of NS5 that alters the PBM motif and hence potentially affecting the affinities and the pattern of cellular PDZ-containing proteins that could be recognized by the NS5 protein during the infection. Interestingly, the substitution of –TVL_COOH_ by –TVM_COOH_ (mPBM) resulted in a significant delay in replication and also in a significantly lower replication with a ten-fold decrease compared to the wt at 48 and 72 h (Fig. 1B) and a five-fold decrease of band intensity for mPBM NS5 compared to wt NS5 as monitored by western blot experiment (Fig. 1C). Collectively, these results show that WNV replication is affected by an amino-acid substitution that alters the PBM within NS5.

Then, we have attempted to look at the knock-down on viral replication of wt WNV and mPBM WNV. We observed viral replication quantified by RTqCR (Suppl. Fig. 2A) and by FFU (Suppl. Fig. 2B). As we did not see any difference for both quantitation, we wondered if any reversions were occurring, permitting this replication. Indeed, after viral amplification, the last residue of the mPBM (Met, ATG) virus had reverted to a Leu (TTG) as the wild-type virus which has a Leu (CTG) (Suppl. Fig. 2C,D). These observations suggest that the Leu in the PBM motif if important for virus amplification.

### West Nile virus NS5 recognizes several cellular PDZ-containing proteins

**Figure 2:**
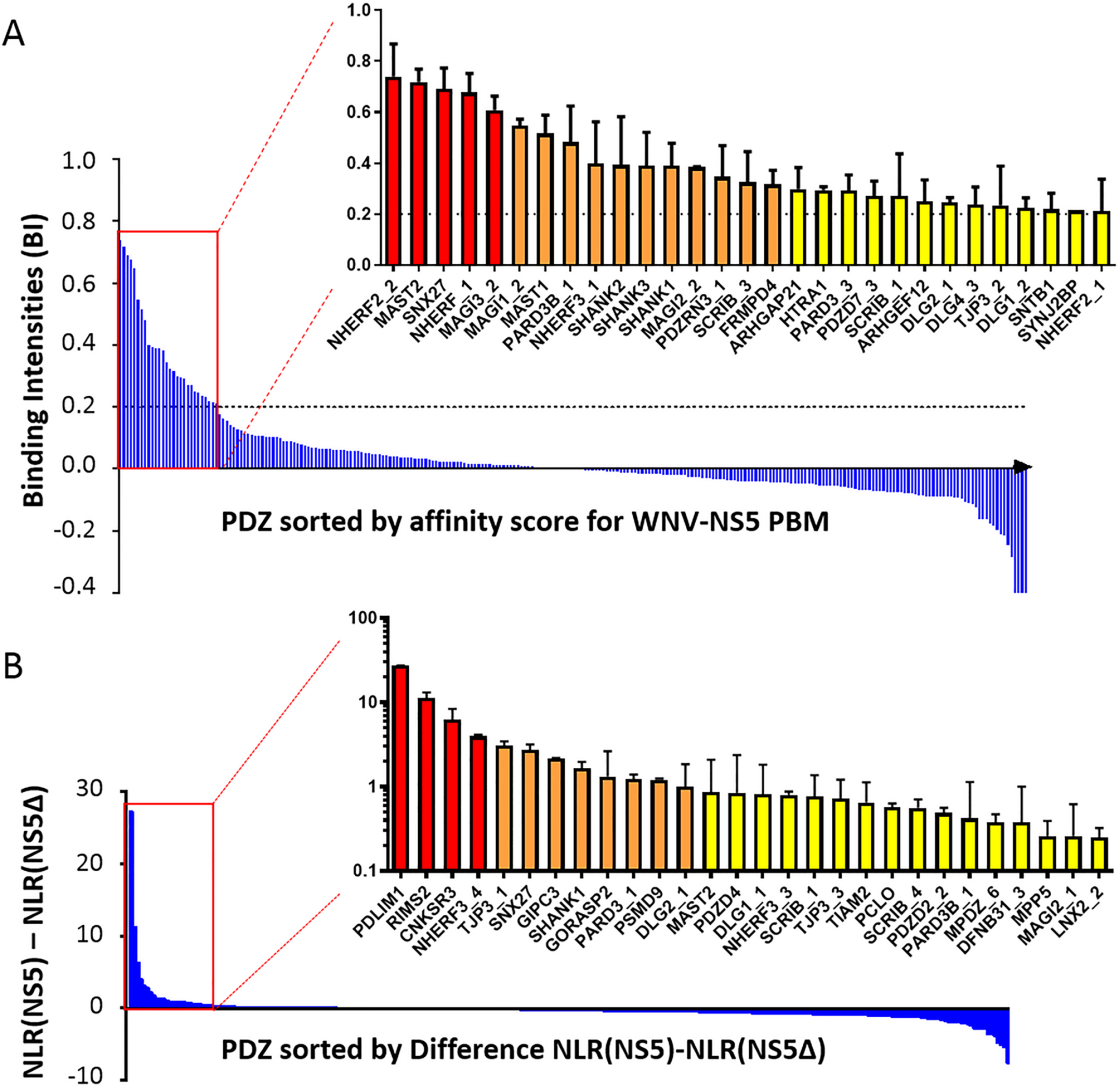
PDZome binding profiles of WNV PBM by two high-throughput techniques: holdup assay (**A**) and Split-Nanoluciferase Protein Complementation Assay (N2H) (**B**). (**A**) Binding intensity profiles for the PDZome. PDZ domains revealed by holdup assay are ranked on the basis of the BIs of WNV NS5 PBM. Zoomed-in view (corresponding to the red box) shows the 29 PDZ NS5 binders which displayed significant BI values higher than 0.2. B. Frequency distribution for NLR(NS5) – NLR(NS5∆PBM) obtained by N2H method are ranked on their values. Zoomed-in view (corresponding to the red box) shows 28 PDZ NS5 binders displayed an NLR difference > 0.25 NLR.

In order to quantify the binding activity of the NS5 PBM we used an *in vitro* automated high-throughput chromatography assay called holdup (Fig. 2A)^14^. A 13-mer peptide was synthetized encompassing the C-terminal PBM sequence of WNV NS5 protein linked to a biotinyl group. This peptide was used as bait to quantify the interaction between the WNV NS5 PBM peptide and a full human PDZ domain library expressed in *Escherichia coli.* The updated PDZ library called PDZome V.2 covers 97.3% of the human “PDZome” (259 over 266 PDZ domains identified in the human genome)^13^. The holdup approach displays a high sensitivity for low-to-medium affinity PDZ/PBM pairs and provides an affinity-based ranking of the identified binders corresponding to a specificity profile. Five replicate experiments were performed. This specificity profile for NS5 PBM with the mean values of binding intensities (BI) is reported in Fig. 2A with an affinity-based ranking. Highest values on the Y-axis indicate the PDZ domains recognized with the best affinities by our viral peptide bait. 29 PDZ NS5 binders displayed significant BI values higher than 0.2, a stringent threshold previously defined for significant binders (Fig. 2A, zoomed-in view, Table 1)^14^. We concluded that the PBM sequence of WNV NS5 protein is unambiguously functional *in vitro* as illustrated by its capacity to bind several PDZ domains in the 1–100 µM affinity range typically found for PDZ/PBM interactions. Thus, the holdup assay identified potential PDZ-domain partners from host that can be classified according to their affinity values for NS5 PBM: 5 PDZ NS5 binders with a BI > 0.6 (NHERF2_2, MAST2, SNX27, NHERF-1 and MAGI_2); 11 PDZ NS5 binders with a 0.3 < BI < 0.6 (MAGI1_2, MAST1, PARD3B_1, NHERF3_1, SHANK2, SHANK3, SHANK1, MAGI2_2, PDZRN3_1, SCRIB_3 and FRMPD4) and 13 PDZ NS5 binders with a 0.2 < BI < 0.3 (ARHGAP21, HTRA1, PARD3_3, PDZD7_3, SCRIB_1, ARHGEF12, DLG2_1, DLG4_3, TJP3_2, DLG1_2, SNTB1, SYNJ2BP and NHERF2_1) (Fig. 2A).

**Table 1:** List of PDZ proteins obtained by the two high-throughput Holdup and N2H methods with their descriptions, their number of PDZ domains, and their values obtained by these two methods (mean ± SD): binding intensity profiles for the PDZome revealed by holdup assay and NLR(NS5) – NLR(NS5∆PBM) obtained by N2H method.

Proteins	Description	PDZ domains	Holdup mean	(NLR_NS5 − NLR_NS5∆) N2H method mean
ARHGAP21	Rho GTPase-activating protein 21	1 domain	0.30 ± 0.09	< 0.25
ARHGEF12	Rho guanine nucleotide exchange factor 12	1 domain	0.25 ± 0.09	< 0.25
CNKSR3	Connector enhancer of kinase suppressor of ras 3	1 domain	< 0.15	6.27 ± 2.09
DFNB31	Whirlin	3 domains	< 0.15	_3: 0.38 ± 0.62
DLG1	Disks large homolog 1	3 domains	_2: 0.22 ± 0.04	_1: 0.82 ± 1.01
DLG2	Disks large homolog 2	3 domains	_1: 0.25 ± 0.20	_1: 1 ± 0.85
DLG4	Disks large homolog 4	3 domains	_3: 0.24 ± 0.07	< 0.25
FRMPD4	FERM and PDZ domain-containing protein 4	1 domain	0.32 ± 0.06	< 0.25
GIPC3	PDZ domain-containing protein GIPC3	1 domain	< 0.15	2.15 ± 0.04
GORASP2	Golgi reassembly-stacking protein 2	1 domain	< 0.15	1.32 ± 1.31
HTRA1	Serine protease HTRA1	1 domain	0.30 ± 0.02	< 0.25
LNX2	Ligand of Numb protein X 2	4 domains	< 0.15	_2: 0.25 ± 0.07
MAGI1	Membrane-associated guanylate kinase, WW and PDZ domain-containing protein 1	6 domains	_2: 0.55 ± 0.03	< 0.25
MAGI2	Membrane-associated guanylate kinase, WW and PDZ domain-containing protein 2	6 domains	_2: 0.38 ± 0.01	_1: 0.26 ± 0.36
MAGI3	Membrane-associated guanylate kinase, WW and PDZ domain-containing protein 3	6 domains	_2: 0.61 ± 0.06	< 0.25
MAST1	Microtubule-associated serine/threonine-protein kinase 1	1 domain	0.52 ± 0.07	< 0.25
MAST2	Microtubule-associated serine/threonine-protein kinase 2	1 domain	0.72 ± 0.05	0.87 ± 1.22
MPDZ	Microtubule-associated serine/threonine-protein kinase 1	13 domains	< 0.15	_6: 0.38 ± 0.09
MPP5	MAGUK p55 subfamily member 5	1 domain	< 0.15	0.26 ± 0.13
NHERF	Na(+)/H(+) exchange regulatory cofactor NHE-RF1	2 domains	_1: 0.68 ± 0.08	< 0.25
NHERF2	Na(+)/H(+) exchange regulatory cofactor NHE-RF2	2 domains	_1: 0.21 ± 0.12 _2: 0.74 ± 0.13	< 0.25
NHERF3	Na(+)/H(+) exchange regulatory cofactor NHE-RF3	4 domains	_1: 0.40 ± 0.16	_3: 0.8 ± 0.08 _4: 3.95 ± 0.19
PARD3	Partitioning defective 3 homolog	3 domains	_3: 0.29 ± 0.06	_1: 1.24 ± 0.16
PARD3B	Partitioning defective 3 homolog B	3 domains	_1: 0.48 ± 0.14 _3: 0.16 ± 0.07	_1: 0.42 ± 0.72
PCLO	Protein piccolo	1 domain	< 0.15	0.57 ± 0.06
PDLIM1	PDZ and LIM domain protein 1	1 domain	< 0.15	27.24 ± 0.22
PDZD2	PDZ domain-containing protein 2	6 domains	< 0.15	_2: 0.49 ± 0.07
PDZD4	PDZ domain-containing protein 4	1 domain	< 0.15	0.85 ± 1.52
PDZD7	PDZ domain-containing protein 7	1 domain	_3: 0.27 ± 0.06	< 0.25
PDZRN3	PDZ domain-containing RING finger protein 3	2 domains	_1: 0.35 ± 0.12	< 0.25
PSMD9	26S proteasome non-ATPase regulatory subunit 9	1 domain	< 0.15	1.21 ± 0.04
RIMS2	Regulating synaptic membrane exocytosis protein 2	1 domain	< 0.15	11.24 ± 1.89
SCRIB	Protein scribble homolog	4 domains	_1: 0.27 ± 0.16 _3: 0.33 ± 0.12	_1: 0.76 ± 0.61 _4: 0.56 ± 0.15
SHANK1	SH3 and multiple ankyrin repeat domains protein 1	1 domain	0.39 ± 0.09	1.66 ± 0.31
SHANK2	SH3 and multiple ankyrin repeat domains protein 2	1 domain	0.39 ± 0.13	< 0.25
SHANK3	SH3 and multiple ankyrin repeat domains protein 3	1 domain	0.39 ± 0.13	< 0.25
SNTB1	Beta-1-syntrophin	1 domain	0.22 ± 0.06	< 0.25
SNX27	Sorting nexin-27	1 domain	0.69 ± 0.08	2.74 ± 0.43
SYNJ2BP	Synaptojanin-2-binding protein	1 domain	0.21 ± 0.01	< 0.25
TIAM2	T-lymphoma invasion and metastasis-inducing protein 2	1 domain	< 0.15	0.65 ± 0.48
TJP3	Zona Occludens 3; Tight Junction Protein 3	3 domains	_2: 0.23 ± 0.15	_1: 3.05 ± 0.40 _3: 0.72 ± 0.49

Proteins	Description	PDZ domains	NLR_NS5S /ORF N2H method	
ARHGAP21	Rho GTPase-activating protein 21	1 domain	4.40	
CNKSR3	Connector enhancer of kinase suppressor of ras 3	1 domain	1.91	
HTRA1	Serine protease HTRA1	1 domain	0.97	
MAGI1	Membrane-associated guanylate kinase, WW and PDZ domain-containing protein 1	6 domains	1.39	
RIMS2	Regulating synaptic membrane exocytosis protein 2	1 domain	0.34	
SHANK2	SH3 and multiple ankyrin repeat domains protein 2	1 domain	4.38	

_1: PDZ domain 1. In the second panel, NLR values obtained by N2H with the full-length proteins containing PDZ domains.

In parallel, we monitored full-length NS5-PDZ domain interactions in a cellular context using the Split-Nanoluciferase Protein Complementation Assay (N2H)^15^. We used the full-length (FL) NS5 protein (wt PBM) instead of NS5 PBM peptide used for holdup assay. We constructed a FL NS5ΔPBM protein which contains –TVL base deletions (∆PBM), used as control. The FL NS5 proteins and the library of PDZ domains (257 over 266 PDZ domains identified in the human genome, as we failed to clone the two PDZ domains DLG5_3 and SNTB2) were cloned in mammalian expression vector for high-throughput protein–protein interaction (PPI) detection^16^. Our new PDZ library in the mammalian vector covers 96.6% of the human “PDZome”. Three replicate experiments were performed. Mammalian vectors containing FL NS5 and PDZome were transfected into 293HEK cells. We monitored PPIs by measuring interaction-mediated normalized luminescence ratio (NLR) for both NS5 and NS5∆PBM. The NLR obtained with FL NS5∆PBM protein, used as negative control, was subtracted from the NLR obtained with FL NS5 (wt PBM) protein and plotted in Fig. 2B. Highest values on the Y-axis indicate the highest intensity of PPI for the NS5 protein containing PBM sequence. 28 PDZ NS5 binders displayed an NLR difference > 0.25 NLR and among them 12 > 1.0 NLR difference (Fig. 2B). Thus, wt PBM in the context of full length NS5 protein is functional *in cellulo*, comforting our *in vitro* results with the holdup assay.

Together, these two high-throughput methods confirmed the functionality of WNV NS5 PBM to bind PDZ-containing proteins. To go further, we started assembling from the human ORFeome collection a subset of ORFs encoding full-length proteins containing PDZ domains to be transferred in the N2H system. To date, our bank covers 60.5% of the human PDZome with 91 full-length proteins out of the 152 human proteins known to contain PDZ domains (Table 1). 23 full-length proteins interacting with full-length NS5 protein displayed an NLR > 1.35 NLR (Suppl. Fig. 1A). We concluded that PBM of NS5 and these PDZ domains were unambiguously functional in this context of full-length proteins.

The three lists of PDZ proteins obtained by high-throughput holdup and N2H methods (PDZ domains and full-length PDZ proteins) were compared using a Venn diagram (Suppl. Fig. 1). Eleven proteins were identified in common by holdup assay and N2H method (PDZ domains), five were identified by holdup assay and N2H method (full length proteins) and four were identified by the two N2H methods (PDZ domains and full-length proteins).

To determine whether a particular ontology class or pathway could be established among the PDZ-containing proteins identified as WNV NS5 binders by our experimental approaches, we chose to use the protein annotation program PANTHER (protein annotation through evolutionary relationships). This overrepresentation test led us to analyze our large-scale experimental data against current annotated gene data set. PANTHER analysis was performed with the two lists of PDZ-containing proteins obtained from holdup (29 PDZ NS5 binders; 27 proteins) and from N2H (28 PDZ NS5 binders; 25 proteins) assays. Protein IDs were imported into the Gene Ontology (GO) enrichment analysis tool based on protein analysis through the evolutionary relationships classification system. The cellular component categories of GO having P values < 0.05 were considered to be statistically significant and then selected. PANTHER overrepresentation test based on the GO of molecular functions, cellular component, biological process and protein class (Suppl. Fig. 1B–D and Suppl. Table 1) showed that the majority of the 41 proteins have binding function, are involved in cellular process and more particularly the top five were annotated to membrane, cell periphery, plasma membrane, cell junction and cell projection. From these 41 proteins, some were already known to interact with viruses, and more particularly with Flaviviruses. Considering the results obtained with our high-throughput approaches, PANTHER analysis and the literature, we selected 10 PDZ-containing proteins for further analysis, which were at least in two lists of proteins identified (Suppl. Fig. 1) and which have at least been documented in interaction with another viral protein: ARHGAP21, CNKSR3, DLG1, HTRA1, MAGI1, PARD3, RIMS2, SHANK2, SNX27 and TJP1 (Table 1).

### Depletion of cellular PDZ-containing proteins affects West Nile virus replication

**Figure 3:**
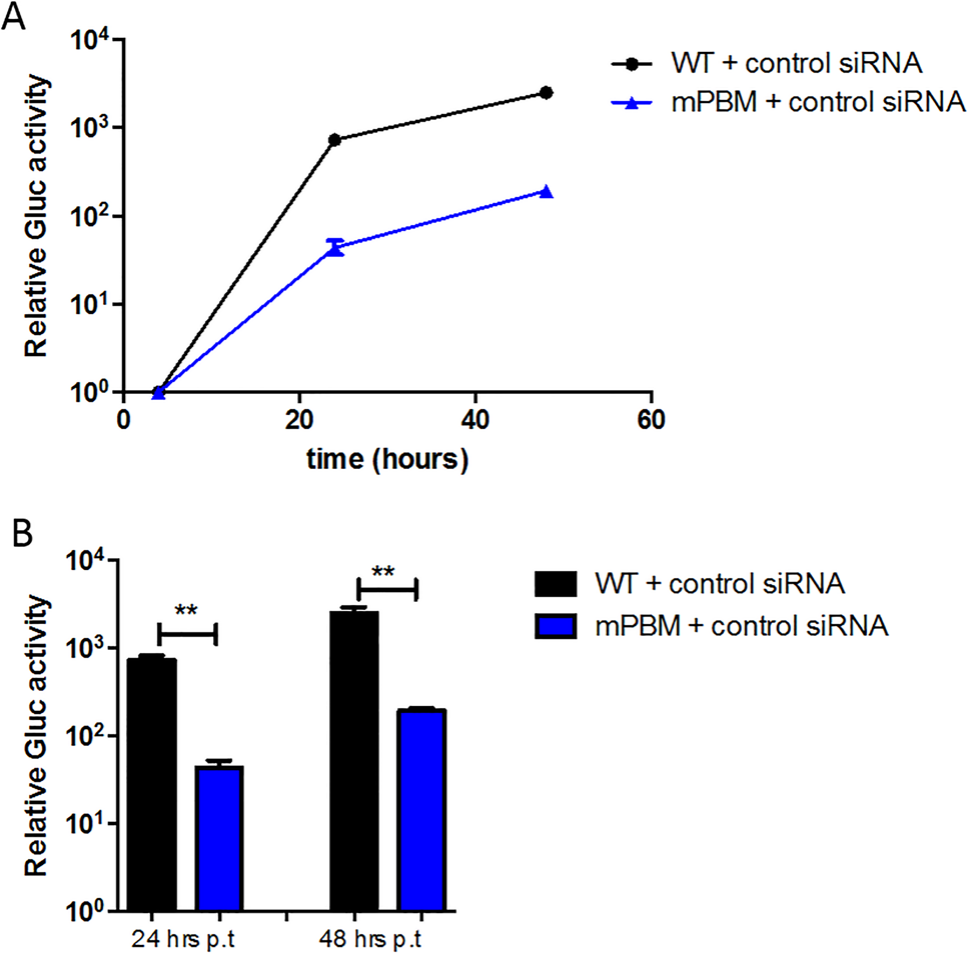
Effect of control siRNA transfection into HEK-293T cells at 24- and 48 h post electroporation of Rep-IS98-Gluc wt and mPBM. HEK-293T cells were transfected with the non-targeting siRNA (control siRNA) 48 h before the electroporation with 10 μg of synthetized WNV replicon RNA. Cells were lysed at 24- and 48 h post electroporation. (**A**) Representative replication curves after electroporation into HEK-293T cells are plotted for Rep-IS98-Gluc-wt (WT) and Rep-iS98-Gluc mPBM (mPBM). (**B**) Statistical data analysis of Rep-IS98-Gluc wt and Rep-IS98-Gluc mPBM activities at 24- and 48 h post electroporation, respectively. Data show relative Gluc activity and error bars represent mean ± SD from three independent experiments in quadruplicate (n = 12). Asterisks indicate significant differences between replicon activities, **p < 0.01.

**Figure 4:**
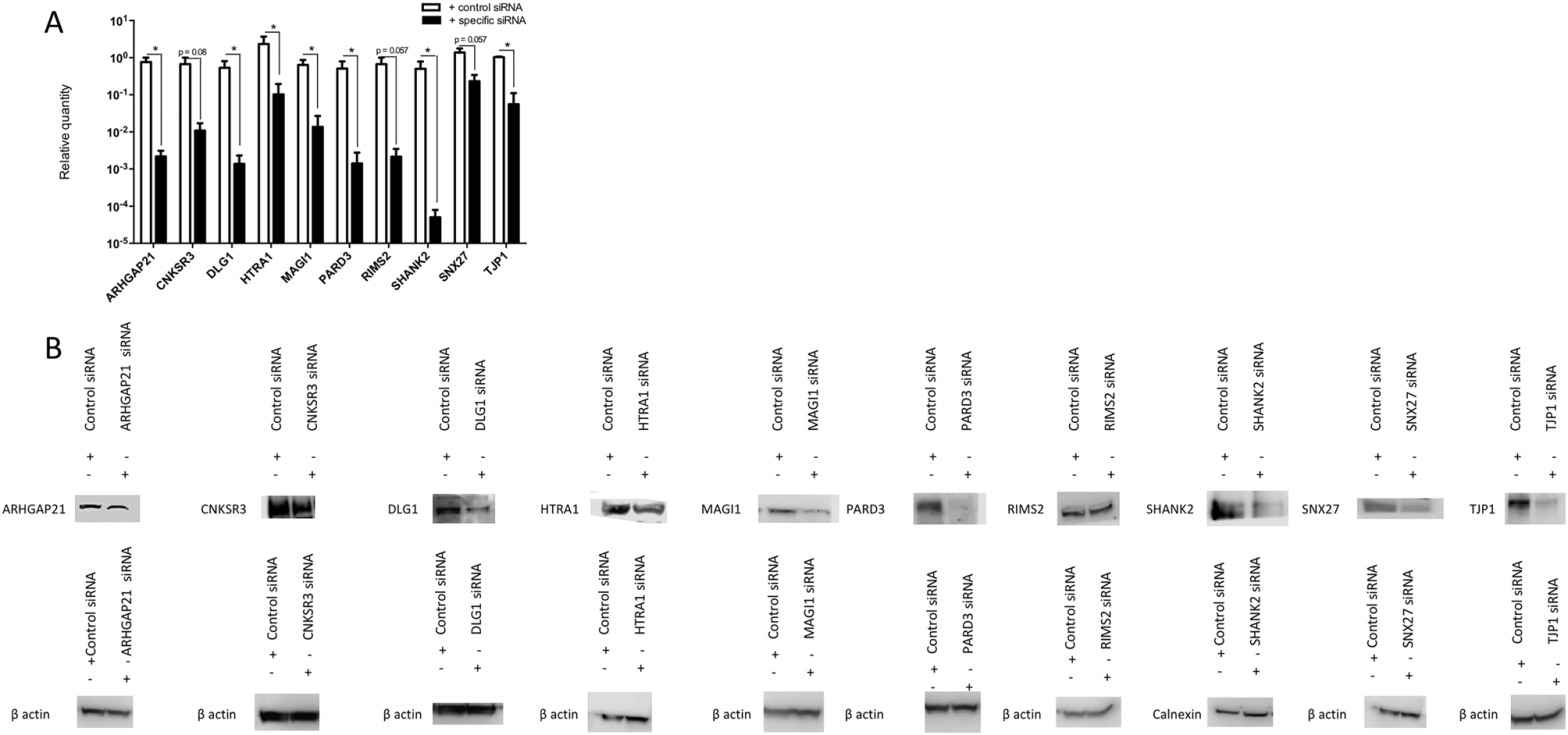
SiRNA knocked down ARHGAP21, CNKSR3, DLG1, HTRA1, MAGI1, PARD3, RIMS2, SHANK2, SNX27 and TJP1 expression. HEK-293T cells were transfected with two siRNAs against each protein: ARHGAP21, CNKSR3, DLG1, HTRA1, MAGI1, PARD3, RIMS2, SHANK2, SNX27 and TJP1 with 10 µM for each target siRNA in Lipofectamine RNAimax. After 48 h, the corresponding transcripts were measured by RTqPCR (**A**) and gene expression measured by Western blot assay (β-actin = loading control) (**B**). (**A**) Total RNA from transfected HEK-293T cells was extracted and used for real time quantitative PCR. Relative expression was normalised to the housekeeping gene GAPDH and is presented as the mean ± SD (*p < 0.05 by Mann Whitney t-test). (**B**) Western blot of a representative sample is showed for each protein. Protein expression was normalized to β actin or calnexin expression and indicated in each WB. Uncropped WB are shown in Supplementary Fig. 3.

To investigate whether the selected cellular PDZ proteins we identified to interact with the WNV NS5 PBM could have a functional role in viral replication, we decided to test depletion of these proteins from HEK-293T cells using siRNAs (Fig. 3). We first compared the replication rate of wt and mPBM replicons with the non-targeted control siRNA using a luciferase assay at different time point over 48 h post-transfection of HEK-293T cells (Fig. 3A). We obtained results similar to that presented in Fig. 1B using BHK-21 cells with a delay in replication for the mPBM (Fig. 3A) and a significant ten-fold decrease compared to wt at 24- and 48-h post-transfection (Fig. 3B). Then, we tested for depletion of the 10 selected cellular PDZ proteins, namely ARHGAP21, CNKSR3, DLG1, HTRA1, MAGI1, PARD3, RIMS2, SHANK2, SNX27 and TJP1 (Table 1). Depletion of the corresponding transcripts was assessed by real-time quantitative PCR and transcripts with a significant decrease in quantity are presented in Fig. 4A. The level of protein expression was checked by Western Blot after siRNA treatment (Fig. 4B). We obtained both a significant decrease in transcripts and a decrease in protein level ranging from 52.5 to 91.6% in HEK-293T cells at 48 h post-transfection (Fig. 4A,B).

**Figure 5:**
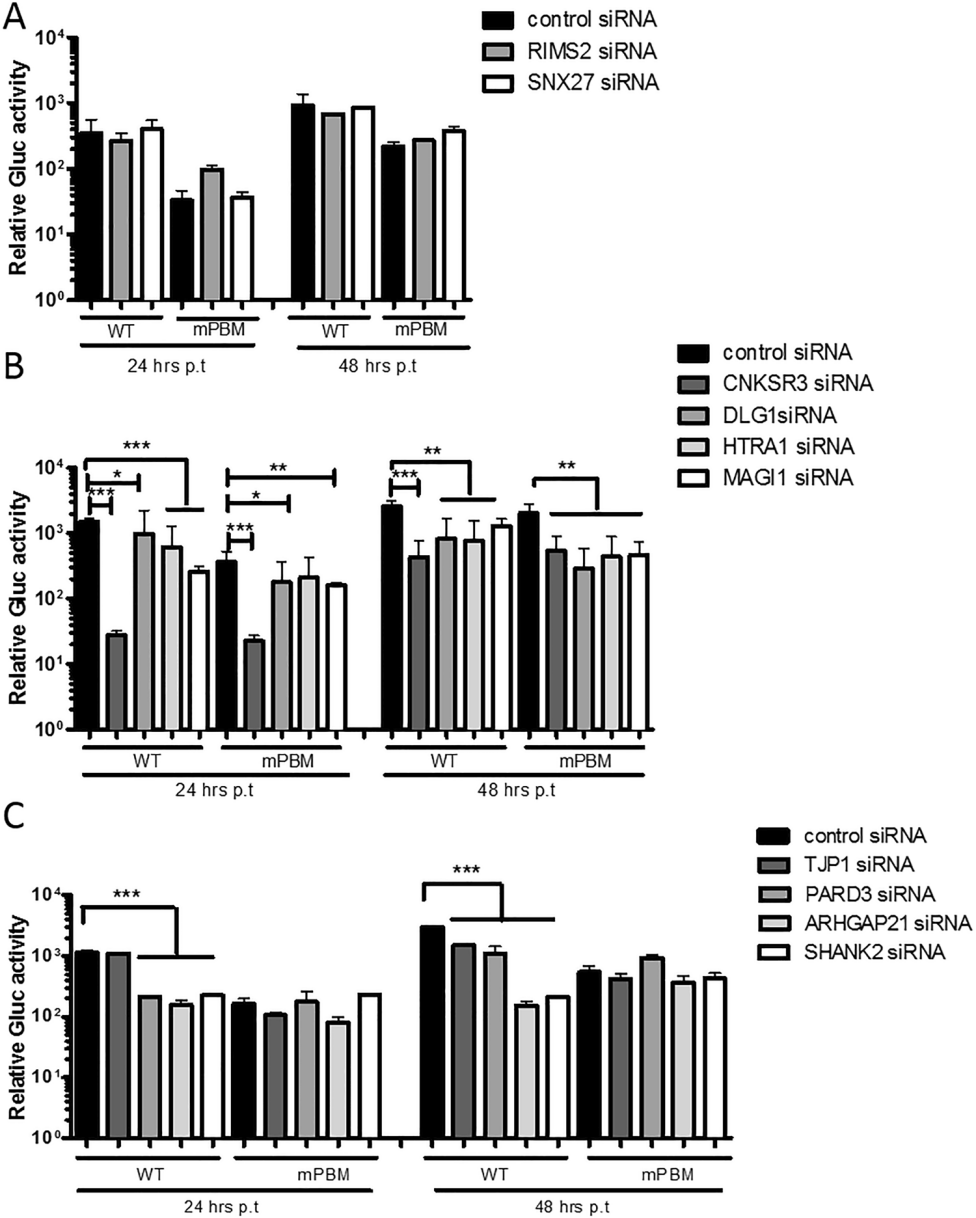
Effect of ARHGAP21, CNKSR3, DLG1, HTRA1, MAGI1, PARD3, RIMS2, SHANK2, SNX27 and TJP1 siRNA transfection into HEK293T cells at 24 and 48 h post electroporation of Rep-IS98-Gluc wt and mPBM plasmids. HEK-293T cells were transfected with ARHGAP21, CNKSR3, DLG1, HTRA1, MAGI1, PARD3, RIMS2, SHANK2, SNX27 and TJP1 siRNA 48 h before the electroporation with 10 μg of synthetized WNV replicon RNA. Cells were lysed at 24- and 48 h post electroporation of Rep-IS98-Gluc wt and Rep-IS98-Gluc mPBM. (**A**) HEK293T cells were transfected with control siRNA, RIMS2 and SNX27 siRNAs showing no effect of the transfection. (**B**) HEK293T cells were transfected with control siRNA, CNKSR3, DLG1, HTRA1 and MAGI1 siRNAs showing an independent-PBM effect. (**C**) HEK-293T cells were transfected with control siRNA, TJP1, PARD3, ARHGAP21 and SHANK2 siRNAs showing a PBM-dependent effect. Data show relative Gluc activity and error bars represent mean ± SD from three independent experiments in quadruplicate (n = 12). Asterisks indicate significant differences between replicon activities, *p < 0.05, **p < 0.01 and ***p < 0.005 obtained with a Mann–Whitney unpaired t-test.

We then monitored the Gluc activity of both wt and mPBM replicons over 48 h post-transfection upon siRNA treatment. Depletion of RIMS2 and SNX27 did not impact replication of either replicon in our conditions at 24- and 48 h post-transfection compared to control siRNA treatment (non-targeted siRNA) (Fig. 5A). RIMS2 and SNX27, although able to interact with WNV NS5 PBM through their PDZ domains *in vitro* and *in cell*, do not seem to play a role in the virus replication in our assay. Depletion of CNKSR3, DLG1, HTRA1 and MAGI1 affected replication of both wt and mPBM replicons in our conditions at 24- and 48 h post-transfection compared to control siRNA treatment, suggesting that these proteins impact WNV replication independently from the substitution we incorporated in the PBM sequence (Fig. 5B). In contrast, depletion of PARD3, ARHGAP21 and SHANK2 resulted in a significant 5.3-, 7.2- and 5.1-fold decrease respectively at 24 h and 2.7-, 19.7- and 14.0-fold decrease respectively at 48 h for the wt replicons post-transfection but not for mPBM replicons (Fig. 5C). Similarly, depletion of TJP1 resulted in a significant two-fold decrease at 48 h of replication for the wt replicons but not for the mPBM replicon. Thus, these results reveal a significant PBM-dependent effect of NS5 on WNV replication involving at least the four host PDZ-containing proteins PARD3, ARHGAP21, SHANK2 and TJP1.

N2H method with full-length proteins (full-length NS5 and full-length human ORF protein) confirmed the interaction between WNV NS5 and four PDZ-containing proteins PARD3, ARHGAP21, SHANK2 and TJP1. Of note, TJP1, PARD3, SNX27 and DLG1 are not present in this bank. We performed a protein–protein interaction in cellular context using N2H method with this new bank and found strong interactions for ARHGAP21 and SHANK2 (NLRs values of 4.41 and 4.38; respectively (Table 1, second part)), in the top 4 over the 91 NLR obtained, we also found a weaker interaction with CNKSR3 (NLR of 1.9). By contrast, RIMS2, HTRA1, MAGI1 displayed low NLR values (Table 1, second part). Thus, we confirmed *in cell* the significant interaction of full length PDZ-containing proteins ARHGAP21 and SHANK2 with the FL NS5 protein.

## Discussion

The viral NS5 protein is the largest and most conserved Flavivirus proteins. It plays a major role in viral replication through its MTase and RdRP domains and in IFN signaling^11,17,18^. Interactions between NS5 PBM and PDZ-containing cellular proteins have been associated with interferon antagonism, viral replication and disease pathogenesis^19–21^. The biological relevance of PDZ binding for TBEV is the most documented, especially regarding viral replication^9–11^. In this study, we showed that the PBM (–TVL_COOH_) motif at the C-terminus of WNV NS5 protein interacts with human PDZ domains *in vitro* but is also capable of interacting with cellular PDZ-containing proteins in a cellular context. We established in this study the promiscuity of 41 different PDZ-containing proteins both *in vitro* and *in cellulo* (listed in Table 1) with the NS5 PBM of WNV by screening a PDZ bank which covers more than 96% of the human “PDZome”. Melik and co-workers previously observed such a broad repertoire of potential host PDZ proteins recognized by the NS5 PBM of WNV using a qualitative PDZ array partially covering the human PDZome^10^. We noted that the lists of partners selected from our two high-throughput screenings only partially overlapped. Several reasons may explain this observation; by contrast with their cellular expression in the N2H assay, the concentrations of partners are strictly controlled *in vitro* holdup assay. While the interactions involved viral PBM peptide and the PDZome in holdup assays, the NH2 screening involves full-length NS5 as bait, the PDZome but also full length PDZ-containing proteins in the case of ORFome screening. Thus, the expression environment and the type of constructs used could also have a strong impact on PPI detection, justifying our choice to combine results from holdup and N2H assays.

Our study supports a short-list of 10 PDZ-containing proteins selected from our crossed high-throughput screenings and for their functions in the cells and during viral infection. We tested the physiological relevance of these 10 interactors on WNV RNA replication using the siRNA technology and the replicon containing an altered PBM with the mutation Leu to Met at the C-terminus of NS5 allowing to conserve a proper predicted RNA structure. Eight of the ten selected PDZ-containing proteins significantly impacted WNV replicon replication: CNKSR3, DLG1, HTRA1, MAGI1, PARD3, ARHGAP21, SHANK2 and TJP1. We show that PARD3, ARHGAP21, SHANK2 and TJP1 displayed a significant PBM-dependent effect on WNV replication using the L-to-M PBM mutation as a gauge of the NS5 PBM influence. In contrast, CNKSR3, DLG1, HTRA1 and MAGI1 affected WNV replication regardless of the presence or absence of mutation in the PBM. We cannot exclude the presence of an unidentified internal PBM in NS5 that might interfere as other PBM motifs located internally rather than at the C-terminus of NS5 were shown to play a role the replication of TBEV and DV^10^. How CNKSR3, DLG1, HTRA1 and MAGI1 proteins are directly or indirectly connected to WNV replication should be documented in further studies. Finally, we identified RIMS2 and SNX27 as WNV NS5 PBM binders. They did not interfere with the viral replication assay, but they could participate in other steps of the viral cycle. Interestingly, RIMS2 has been shown to interact with TBEV NS5, potentially affecting the neuronal signalling of this neurotropic Flavivirus^9^.

Importantly, all our selected PDZ-containing proteins, except CNKSR3 and SHANK2, have been previously reported as host proteins targeted by viruses. MAGI1, PARD3, RIMS2 and TJP1 are targets of Flavivirus proteins (WNV, TBEV and DV)^9–11^. Others cellular PDZ proteins were also identified as target for other virus including DLG1, MAGI1 and ARHGAP21 for Influenza virus^16,22–24^; HTRA1, PARD3, SNX27, DLG1, MAGI1 for Human Papillomavirus^11,25–28^. The large number of cell proteins targeted by WNV could be related to the broad host repertoire of Flaviviruses replicating in very different cells and species necessary for its complex zoonotic transmission cycle. Our PANTHER analysis shows that these ten proteins were involved in a global perturbation of cellular homeostasis, architecture of the cells (membrane trafficking, cell junction, cellular polarity…), innate immune response (IFN mediated-JAK-STAT signalling) and neuronal cells (synapse). We report that NS5 of WNV can interact with TJP1 (also known as ZO-1), ARHGAP21 and PARD3 proteins through PBM-PDZ interactions. These three proteins are involved in cell polarity, trafficking, cell adhesion and cell junction. ARHGAP21 protein is involved in trafficking through the control of CDC42 activity^29^. Taye *et al. *performed a microarray analysis and obtained an overexpression of ARHGAP21 which negatively regulates the transport of Influenza A virus (H1N1) neuraminidase to the cell surface^22,30^. TJP1 was reported to interact with other Flavivirus NS5^9,10^. Interestingly, we found both TJP1 and TJP3 as interactors of WNV NS5 PBM in our study (Fig. 2A and B and Table 1). TJP1, TJP2 and TJP3 are members of the membrane-associated guanylate kinase (MAGuK) family of proteins^31^ that play a particular role in tight-junctions (TJs) organization^32^. TJP1 can bind directly to TJP3 to form a TJP1/TJP3 complex that acts directly on the actin skeleton. Interestingly, it was demonstrated that actin filaments participate in WNV maturation process^33^. Moreover, TJs assembly and permeability are highly regulated by signalling pathways^34^. It was shown that TBEV NS5 interacts with GIPC and TJP2 through PDZ/PBM complex. The MAGuK TJP2 protein is an interactor of Scribble, a PDZ-containing protein involved in cell polarity and neuronal function. The complex NS5-TJP2-hScrib may shuttle the polymerase to the plasma membrane, regulating the flaviviral replication that occurs at ER membrane-associated complexes^11^. As TJP1, PARD3 displayed a significant NS5 PBM-dependent effect on WNV replication. Chen *et al.* showed that suppression of PARD3 expression by RNA interference (RNAi) caused a dramatic disruption of TJ assembly^35^. Of note, we also detected PARD3B, a human PARD3 homologue (Fig. 2A,B and Table 1), which also localizes at the TJs^36^. Moreover, PARD3 and TJP1 are recruited by JAM-2 to cell–cell contacts^37^. TJP1 is also associated with JAM-1 through PDZ domain 3^38^. The association of TJP1 with these different proteins in TJs serves to cluster the integral membrane proteins at TJs and could be partially disrupted by WNV NS5 during infection resulting in a progressive disruption of TJ, which leads to leakage between adjacent cells, loss of barrier function, and infiltration of WNV virions^37^.

Suppression of host innate immunity is one of the multiple crucial functions of Flavivirus NS5 protein. The type I IFN signalling pathway included in the first lines of defence against Flavivirus infection of mammals. TBEV and WNV NS5 inhibit IFN-I signalling by suppressing surface expression of interferon alpha and beta receptor subunit 1 (IFNAR1)^39^. Indeed, TBEV may manipulate the IFN pathways through interaction with TJP1 as TBEV-NS5 (and DV-NS5) was shown to be present in the nucleus at the same moment as TJP1^9^. To note, in WNV-infected cells, a high proportion of NS5 resides in the nucleus^40^ but it is also accurate for other Flavivirus NS5 (DV^41^; Yellow Fever^42^). A consequence of the NS5/PDZ-containing protein interactions could be a mislocalization of these PDZ-containing proteins which can alter their original function. For example, TJP2 and MAGI1 were reported to be sequestered by adenovirus E4-ORF1 in the cytoplasm of fibroblasts^43^ and this mislocalization disrupted both the tight junction barrier and the apicobasal polarity.

The PDZ-containing proteins interaction with NS5 protein could result in a less restrictive environment for viral replication and provide a new understanding on the mechanism of WNV immune evasion.

Several of the PDZ-containing cellular proteins identified in our crossed high-throughput screenings are involved in the development, function and architecture of neuronal cells (RIMS2, SHANK2, DLG1), highlighting the relevance of our findings as WNV is indeed a neurotropic virus. The nature of the assay we used, restricted the characterization of the functional significance of such PDZ dependent WNV-host interactions to viral replication. Further characterization of the mechanisms by which PBM/PDZ interactions may alter neuronal functions is therefore essential.

## Materials and methods

### Cell lines and viruses

Baby Hamster Kidney fibroblasts (BHK-21) cells and human embryonic kidney 293 T (HEK-293T) cells were grown and maintained in Dulbecco's Modified Eagle Medium (DMEM) supplemented with 5% fetal bovine serum (FBS, Qualified, Gibco) and in DMEM supplemented with 10% FBS and 100 U ml^−1^ penicillin, 100 U ml^−1^ streptomycin, respectively. Both lineages were cultured at 37 °C in a humidified 5% CO_2_ incubator. The replicon Rep-IS98-Gluc/pCR2.1 has been previously described^30^.

### Production of recombinant WNV

We first produced a full-length infectious clone for wt WNV and mPBM WNV as previously described by Alsaleh *et al.*^44^; the DNA was purified and transcribed *in vitro* using the mMessage SP6 kit (Thermo Fisher Scientific). The resulting RNAs were electroporated in C6/36 cells (400 V, 25 µF, 800 Ω) in OPTI-MEM medium (Thermo Fisher Scientific). Cell culture supernatants were collected at 72 h post-electroporation and used to infect 10^7^ C6/36 cells. At 3 days post-infection, viral supernatants were amplified by infecting 5 × 10^7^ C6/36 cells for 3 days before collection and utilization as final viral stocks. The two stocks were used for genome quantitation by RTqPCR and for virus titers by FFU using Vero cells. Full-length viral genomes were sequenced by Sanger method from cDNA obtained by reverse transcription using the Superscript II reverse transcription kit (Invitrogen) and amplified by PCR using the Phusion high-fidelity kit (Thermo Fisher Scientific).

### Cloning of human PDZ domains for Holdup assay

The full PDZome clone collection (266 clones), previously cloned by Vincentelli *et al*.; PDZome V.2^16^. These constructs were initially cloned into pDONRzeo for the 266 clones. All the PDZ (266 clones) were sequenced in the destination vector.

### Holdup assay with human PDZ domains

All PDZ domains (259/266 PDZ) were expressed following the high-throughput protocol previously described^45^. The peptides biotinylated WNV NS5 PBM (peptide sequence-RYEDTTLVEDTVL) was synthesized in solid phase using Fmoc strategy (Proteogenix) and resuspended in H_2_O. The holdup assay was carried out against the biotinylated WNV NS5 PBM in quadruplicates as previously described^14,45^ with minor modifications. We measured WNV NS5 PBM interactions against 259 human PDZ domains. The minimal BI threshold value is 0.2 to define a significant interaction as previously reported^14^.

### Cloning all PDZ domains and ORFs into gateway-compatible expression plasmids for split-nanoluciferase protein complementation assay (N2H)

All PDZ domains (257/266 PDZ) and ORFs containing PDZ domains (91 full-length proteins out of the 152 human proteins) were introduced into Gateway destination vector pYN2H-LEU-N1^45^ whereas NS5 and NS5∆PBM (NS5 with a deletion of the three residues –TVL–) were introduced into Gateway destination vector pYN2H-TRP-N2, via LR clonase-mediated Gateway reaction (Life Technologies), following the protocol previously described^46^. Briefly, LR reaction products were subsequently transformed into DH5α competent bacterial cells and grown for 24 h at 37 °C at 900 rpm in carbenicillin-containing TB liquid medium. Plasmid DNA was extracted using a NucleoSpin 96 Plasmid kit from Macherey–Nagel, following manufacturer's instructions. All DNA were sequenced using plasmid-specific primers.

### Split-nanoluciferase protein complementation assay (N2H) into HEK cells

HEK-293T cells were seeded at 5.4 × 10^4^ cells per well in 96-well, flat-bottom, cell culture microplates (Greiner Bio-One, #655083), and cultured in Dulbeccoʼs modified Eagleʼs medium (DMEM) supplemented with 10% fetal calf serum at 37 °C and 5% CO_2_. Twenty-four hour later, cells were transfected with 100 ng of pYN2H-LEU-N bait and 100 ng of pYN2H-TRP-N2 prey using linear polyethylenimine (PEI) to co-express the peptide/protein pairs or the protein/protein pairs fused with complementary NanoLuc fragments, N1 and N2, as previously described by Choi *et al.*^46^. Twenty four hours after DNA transfection, the cell culture medium was removed and 50 µL of mix (100 mM MES pH 6.0, 1 mM CDTA, 0.5% (v/v) Tergitol, 0.05% (v/v), Mazu DF 204, 150 mM KCl, 1 mM DTT, and 35 mM thiourea; Furimazine 20 µg/ml) were added to each well, plates were incubated for 3 min at room temperature, and luminescence output was measured using a Centro XS LB 960 luminometer (Berthold; 2 s integration time). All binary PPI experiments were independently performed three times. Control experiments were performed similarly where NS5/NS5∆PBM protein fused to the NanoLuc fragment N1 was co-expressed with the matching NanoLuc fragment alone. For each peptide/protein pair –N1/–N2, we calculated a normalized luminescence ratio (NLR) corresponding to the raw luminescence value of the tested pair (–N1/–N2) divided by the maximum luminescence value from the control, as previously described by Cassonnet *et al*.^15^. One NLR was obtained for the NS5/PDZ and one for NS5∆PBM/PDZ. We decided to subtract the NLR calculated with NS5∆PBM protein to the NLR calculated with NS5 protein, all the values are listed in the Table 1 with mean ± SD.

### Plasmid DNA constructs

All recombinant DNA techniques and cloning procedures were carried out by standard procedures^47^. We modified the replicons by site-directed mutagenesis methods on the PBM of NS5 proteins using Phusion polymerase the following primers respectively: Forward mPBM: 5′GGTTGAGGACACAGTAATGTAGATATTT3′, Reverse mPBM: 5′AAATATCTACATTACTGTGTCCTCAACC3′.

### *In vitro* transcription

The RNA was transcribed from linearized reconstituted full-length plasmids using mMESSAGE MEGAscript Sp6 Kit (Ambion-Life technologies) according to the manufacturer’s instructions as previously described^44^. Briefly, after 4 h of incubation at 37 °C, the DNA template was digested by DNase treatment. Synthesized RNA was precipitated by LiCl and purified according to manufacturer’s instructions (Ambion-Life Technologies). The RNA was quantified by Nanodrop and used for transfection.

### Plasmid transfection into BHK-21 and HEK-293T cells and Luciferase assays

4 × 10^6^ BHK-21 cells were electroporated with 10 μg of synthetized RNAs using the following settings: 1 pulse of 140 V, 25 µF and ∞ Ω. Cells were resuspended in DMEM-2% FBS and seeded at 1 × 10^5^ cells/well in a 24 well plate.

4 × 10^6^ HEK-293T cells electroporated with the different siRNAs were electroporated with 10 μg of synthetized RNAs using the following settings: 1 pulse of 220 V, 960 µF and 25 ms. Cells were resuspended in DMEM-10% FBS and seeded at 2 × 10^5^ cells/well in a 24 well plate.

Cells were lysed at 4-, 24-, 48- and 72 h post electroporation with Renilla Luciferase Assay Lysis Buffer according to the manufacturer’s instruction (Promega). Mean values of Renilla-normalized firefly Luciferase expression were determined from quadruplicate wells. At least three independent transfections in cells were performed for each experiment.

### siRNA

Two siRNAs against each protein: ARHGAP21, CNKSR3, DLG1, HTRA1, MAGI1, PARD3, RIMS2, SHANK2, SNX27 and TJP1 (Suppl. Table 2) were purchased from Life Technologies (10 μM for each target siRNA and 20 μM for control untargeted siRNA) were transfected into HEK-293T cells (24 well cultures dishes) using Lipofectamine RNAimax according to the manufacturer's reverse-transfection protocol. After 48 h, the cells were used for electroporation of RNA replicons as described above. Data were analyzed from two experiments in quadruplicate wells.

### RTqPCR

Total RNA from infected or transfected HEK-293T cells was extracted using Nucleospin RNA kit (Machery Nagel) according to the manufacturer's protocol and reverse transcribed into cDNA using Moloney Murine Leukemia Virus Reverse Transcriptase (Invitrogen, Life Technologies). A SYBR Green-based real-time PCR assay (QuantiTect SYBR Green kit, Qiagen) for relative quantification of transcripts was performed on 384-well plate QuantStudio 6 Flex real-time PCR system (Applied Biosystems) according to protocol as previously described^19,20^. The relative quantities were determined by using raw Cp values as input for qBase, a flexible and open source program for qPCR data management and analysis^48^; the primers used in this manuscript were listed in Suppl. Table 2.

### Western Blot

Protein lysates were prepared by cell lysis in radioimmunoprecipitation assay (RIPA) buffer (BioBasic Canada) containing protease inhibitors (Roche), then the cells were frozen at −20 °C. Five μl of NuPAGE LDS sample Buffer (Thermo Fisher Scientific) was added to 20 μl of lysate and incubated at 95 °C for 5 min. Samples were separated on NuPAGE 4–12% Bis–Tris Protein Gels and transferred to a polyvinylidene difluoride (PVDF) membrane (Life Technologies). The membrane was washed for 1 h at room temperature in PBS-Tween (PBS-T) plus 5% milk, then incubated overnight at 4 °C with the different antibodies (Suppl. Table 2). After washing in PBS-T the membrane was incubated for 1 h at room temperature with HRP-conjugated antibody (BioRad). After washes in PBS-T, the membrane was developed with enhanced chemiluminescence reagent (Pierce) and exposed to film.

### Statistical analysis

The *p* values were determined with GraphPad Prism software version 8.0 (https://www.graphpad.com/scientific-software/prism/) using a Mann–Whitney unpaired t-test (*p < 0.05, **p < 0.01, ***p < 0.005).

## Supplementary Information

Supplementary Information.
