# Supplementary material for: Role of PDZ-binding motif from West Nile virus NS5 protein on viral replication
Source: Sci Rep. 2021 Feb 5;11:3266. doi: 10.1038/s41598-021-82751-x (PMC7865074; doi:10.1038/s41598-021-82751-x)
Supplement: Supplementary file 1 — Supplementary Information. [file 41598_2021_82751_MOESM1_ESM.docx]

**Supplementary materials**

**Role of PDZ-binding motif from West Nile virus NS5 protein on viral replication.**

**Authors:**

Emilie Giraud^1*^, Chloé Otero del val^2^, Célia Caillet-Saguy^2^, Nada Zehrouni^2^, Cécile Khou^5^, Joël Caillet^4^, Yves Jacob^3^, Nathalie Pardigon^5^, Nicolas Wolff^2^.

^1^ Unité Interactions Virus-Insectes, Institut Pasteur, Paris.

^2^ Unité Récepteurs-Canaux, Institut Pasteur, Paris.

^3^ Unité de Génétique Moléculaire des virus ARN, Institut Pasteur, Paris.

^4^ UMR 8261, CNRS, Université de Paris, Institut de Biologie Physico-Chimique, 75005, Paris, France.

^5^ Unité de Recherche et d’Expertise Environnement et Risques Infectieux, Institut Pasteur, Paris

* Corresponding author: Emilie Giraud

E-mail: [emilie.giraud@pasteur.fr](mailto:emilie.giraud@pasteur.fr)

**Keywords:**

Flavivirus, Virus replication, Viral protein, protein-protein interaction, PDZ domain, PBM motif.

**Contents**:

Supplementary Figures 1-3 and supplementary tables 1-2

**Suppl. Fig. 1: Functional categorization of PDZ proteins identified by the high-throughput Holdup and N2H methods**

(A) Venn diagram comparing the list of PZD proteins obtained by Holdup (blue), by N2H method with the PDZ domains (red) and by N2H method obtained with full-length proteins (green). (B-E) Pie charts of PANTHER GO-slim molecular function (B), Biological process (C), Cellular function (D) and protein class (E). PANTHER overrepresentation test was performed on 41 proteins which were identified by the two high-throughput analysis by importing protein IDs into the GO enrichment analysis tool based on protein analysis through the evolutionary relationships (PANTHER) classification system.


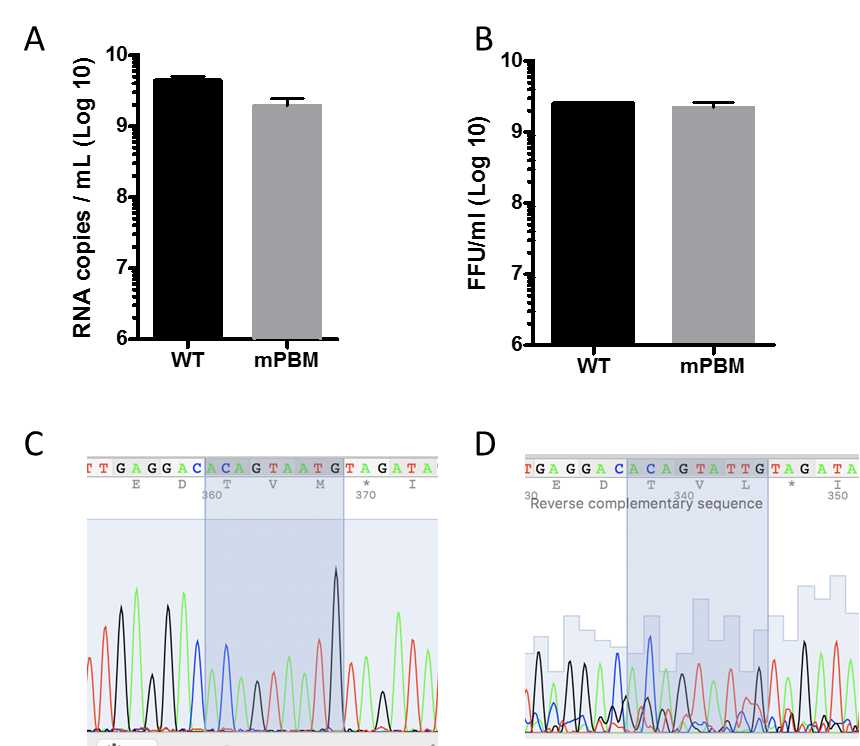


**Suppl. Fig. 2: Characterization of recombinant wt and mPBM WNV *in vitro*.**

(A) Viral stocks of wt and mPBM WNVwere quantified by viral genome quantitation by RTqPCR and (B) by virus titers by FFU using Vero cells. Representative Sanger sequence chromatograms from wt WNV (C) and mPBM WNV (D) of both viral stocks showed that the mutation Met (ATG) reverted into a Leu (TTG); another codon for Leu than the one in wt WNV (CTG).


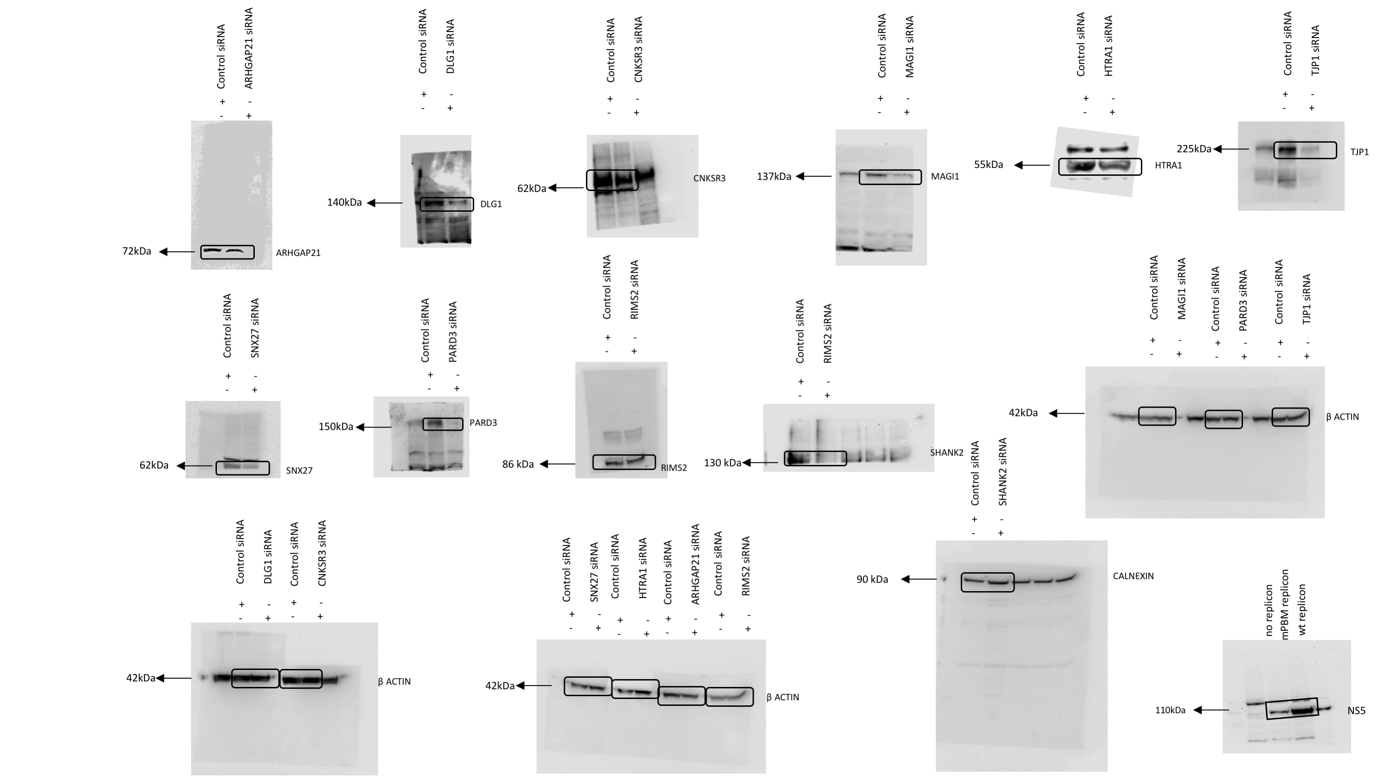


***Suppl. Fig. 3: Uncropped Western Blots4B and identification of band size from Figure 1 (A.) for NS5 and from Figure 4B for the siRNA knocked down ARHGAP21, CNKSR3, DLG1, HTRA1, MAGI1, PARD3, RIMS2, SHANK2, SNX27 and TJP1 expression.*** *For all Western Blots, membranes were used for several samples and were all cut in several parts prior to hybridization with antibodies.*

Suppl. Table 1: The reference list contains all proteins in Homo sapiens. PANTHER overrepresentation test was performed on 41 proteins which were identified by the two high-throughput analysis by importing protein IDs into the GO enrichment analysis tool based on protein analysis through the evolutionary relationships (PANTHER) classification system. The statistical comparisons were made by Fisher’s exact test with Bonferroni correction.

Suppl. Table 2: supplementary informations for i) siRNA, ii) RTqPCR and iii) Western blot.

RbM: rabbit monoclonal; MM: mouse monoclonal; RbP: rabbit polyclonal.
